# Recurrent dedifferentiated liposarcoma with histological grade progression: a case report

**DOI:** 10.3332/ecancer.2025.1831

**Published:** 2025-01-22

**Authors:** Samuel Santiago Parra Giraldo, Rut Amparo Vergara López, Haydee De La Hoz-Herazo, Enrique Carlos Ruiz Pla, Brayan Bayona-Pacheco, Juan Jose Espitia De La Hoz

**Affiliations:** 1 Division of Health Sciences, Department of Medicine, Universidad del Norte, Barranquilla 081007, Colombia; 2 Unidad de Patología Diagnostica UPC, Barranquilla 080001, Colombia; 3 Centro Especializado en Radiología e Imágenes Diagnósticas CERID, Barranquilla 080001, Colombia; 4 Division of Health Sciences, Department of Public Health, Universidad del Norte, Barranquilla 081007, Colombia

**Keywords:** Dedifferentiated liposarcoma, immunohistochemistry, recurrence, soft tissue sarcoma

## Abstract

**Introduction:**

Dedifferentiated liposarcoma (DDLPS) is a rare mesenchymal neoplasm that accounts for approximately 20% of soft tissue sarcomas in the human body. This case report emphasises a high-grade DDLPS with a retroperitoneal location and its unexpected recurrence in a 72-year-old male patient more than 10 years after the primary tumour. This case is particularly significant because of the anomalous presentation of the tumour recurrence time that is complemented by the unusual histologic features of the initial neoplasm, which raises new questions about the biological behaviour of the disease, the clinical course and the management of this pathology.

**Expected results:**

The report seeks to highlight the important and unusual aspects of the pathology that can contribute to a better understanding of its evolution, allowing informed clinical decisions to improve the patient’s quality of life and prognosis.

## Background/Introduction

Soft tissue sarcomas are neoplasms of mesenchymal origin that correspond to approximately 1% of all types of cancer. Sarcomas have more than 50 histological subtypes, including liposarcoma (LPS), which accounts for 20% of soft tissue sarcomas, which in turn are subdivided according to their histopathological characteristics, including dedifferentiated liposarcoma (DDLPS) [1]. Macroscopically, DDLPS is characterised by a multinodular appearance, yellow-grayish colouration and extensive areas of necrosis. Histopathologically, the presence of spindle-shaped, hyperchromatic cells with marked pleomorphism with elongated nuclei and giant cells was observed. In addition, there is the occasional appearance of a multi-vacuolated lipoblast. This heterogeneous nature and its poorly differentiated morphology represent a clinical challenge for diagnosis and prognosis.

DDLPS is a rare variant of LPS, which is characterised by mainly affecting middle-aged and older adults. The incidence of DDLPS is approximately 0.1 cases per million people during each year [2]. At the immunohistochemical level, the overexpression of genes such as *murine double minute 2* (MDM2), *Cyclin dependent kinase 4* (CDK4), *Cyclin dependent kinase inhibitor 2* (P16) and vimentin in the multiple dedifferentiated zones are highly sensitive markers for the diagnosis of DDLPS, allowing us to differentiate it from other types of soft tissue sarcoma [3]. The clinical picture of DDLPS is variable. Some patients present with nonspecific symptoms such as pain and peripheral edema, whereas others may remain asymptomatic until further progression of the disease. In some cases, compression or invasion of adjacent structures may result in more severe and complex symptoms. The main treatment is surgical resection of the tumour, considering radiotherapy and chemotherapy in those metastatic tumours or unresectable.

## Methods

Case report. Year 2023. Carried out at the Clinical Pathology Unit, Barranquilla.

### Presentation of the case

A 72-year-old male patient with a significant oncologic history of diagnosis more than 10 years ago of a low-grade DDLPS located in the right inguinal region that invaded the ipsilateral iliofemoral vessels, which debuted with swelling in the lower abdomen and edema of the right lower limb. Exploratory laparotomy was performed with total resection of the tumour and arteriovenous grafting of the involved vessels.

**Figure 1.**
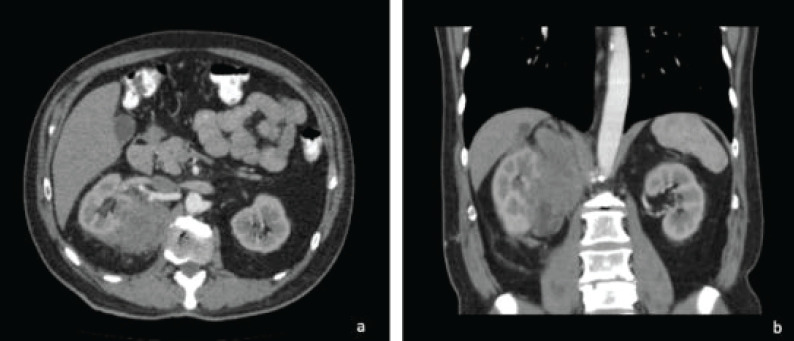
Axial (a) and coronal (b) section of abdominal tomography with contrast. There is a mass with heterogeneous enhancement, irregular borders, retroperitoneal location, infiltrating the upper pole of the right kidney.

Ten years after the initial picture, during a control abdominal ultrasound, the presence of an abdominal mass of right retroperitoneal predominance was evidenced, compatible with the clinical diagnosis of a malignant tumour, probably sarcoma, involving the adjacent renal parenchyma. At the consultation, the patient was asymptomatic. In view of these new findings, a CT scan was requested (Figure 1).

The tomography confirmed the presence of a right retroperitoneal mass of 17 × 9 cm of maximum axes, which invaded the right renal parenchyma and extended toward the upper abdominal region contacting the ipsilateral diaphragm and the right psoas.

**Figure 2.**
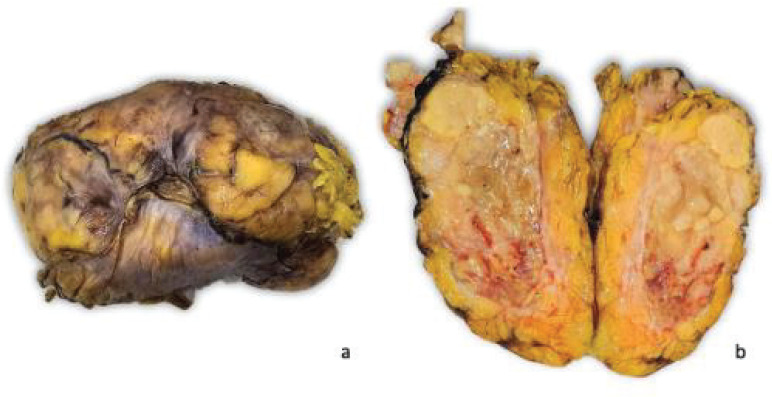
Macroscopic findings. The specimen presents multilobulated aspect and on section the tumour is heterogeneous with extensive areas of necrosis that cause loss of the usual renal architecture.

Right radical nephrectomy plus resection of the retroperitoneal mass was performed. The specimen was sent for an anatomopathological study. Macroscopically, the specimen weighed 860 g and measured 17 × 9 × 8 cm. Externally, it presents a congestive and multilobulated aspect. Serial sections showed a yellowish colouration, heterogeneous aspect, with necrotic areas and firm consistency. The tumour infiltrated a large part of the right kidney, without the involvement of the renal hilum or the ipsilateral adrenal gland (Figure 2).

**Figure 3.**
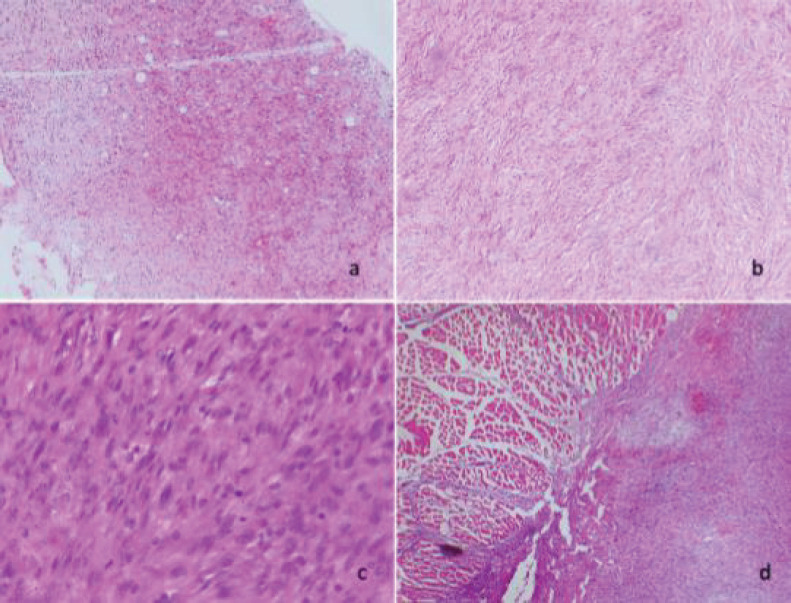
(a) and (b): Diffuse and discohesive proliferation of spindle cells (4× and 10×). c) Tumour cells show moderate nuclear pleomorphism and occasional mitoses (40×). (d): Infiltration of neoplastic cells into the skeletal muscle of the diaphragm (40×).

Microscopically, diffuse cellular proliferation of spindle-shaped appearance with elongated, hyperchromatic nuclei, with moderate nuclear pleomorphism and extensive areas of necrosis was evidenced. The tumour cellularity is intermingled with some lobules of adipose tissue. In other areas, lipoblast is observed. The lesion is focally infiltrating the diaphragmatic muscle tissue (Figure 3).

**Figure 4.**
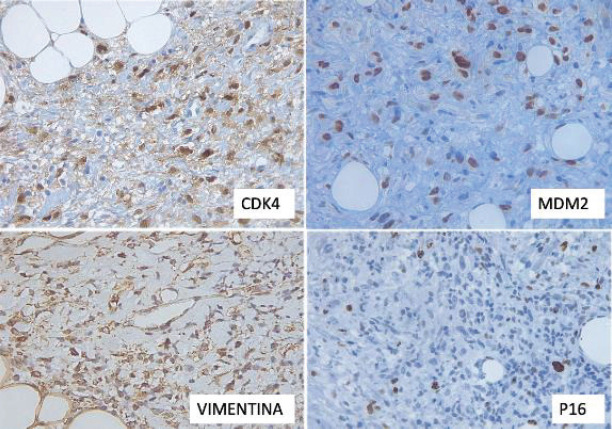
Immunohistochemical findings. Tumour cells were positive for CDK4, MDM2, Vimentin and p16.

Immunohistochemistry (IHC) revealed positivity in the tumour cells for vimentin, P16, CDK4 and MDM2, with a proliferative index (Ki-67) of approximately 15%. AML, S100 and CD99 were negative (Figure 4).

## Results and discussion

IHC has innovated the way pathologists diagnose and classify neoplasms, providing an accurate tool for the identification of both cellular and molecular features. In DDLPS, a rare malignancy, IHC is crucial in its detection and differentiation from other tumour processes. Molecular analysis has shown that DDLPS often has amplifications on chromosome 12q13-15, including oncogenes such as MDM2, which has a 100% sensitivity for differentiation from other benign lipomatous tumours and the CDK4 gene, which is essential for differentiation from other tumours that mimic the histopathologic confirmation of DDLPS [3]. These genes play crucial roles in cell cycle regulation and apoptosis, thus contributing to their aggressive behaviour. The participation of the MDM2 gene in the pathophysiology of the disease is determinant since this gene encodes an E3 ubiquitin ligase that negatively regulates the tumour suppressor p53, inhibiting apoptosis and promoting cell proliferation. For its part, CDK4 forms a complex with cyclin D that has the ability to phosphorylate the retinoblastoma protein. This phosphorylation promotes the cell cycle progression. When CDK4 is overexpressed, the cell cycle progression becomes uncontrolled, contributing to excessive cell proliferation and disease development. These genetic alterations not only favor carcinogenesis, but also correlate with the aggressiveness of the tumour and its degree of dedifferentiation [4]

Recurrence of DDLPS is a relevant feature and is associated with multiple factors, including the quality of surgical resection of the tumour, the molecular conformation and the histological grade. In this case, the dedifferentiated histologic subtype was generally high grade with a high rate of aggressiveness [5] which confers a higher probability of local recurrence and distant metastasis. Local recurrence of these tumours is expected within 5 years following the initial treatment [6]. Distant metastasis is associated with a worse prognosis, primarily in DDLPS that form de Novo rather than in those that develop from a well-differentiated LPS [7]. The combination with local recurrence greatly decreases survival, and it is necessary to specify that the most common site is the lung/pleura, followed by subcutaneous and/or intramuscular tissue [8, 9]. The recurrence of these tumours 10 years after resection of the primary tumour is not usual; however, this case provides us with valuable information that orients us on the heterogeneous behaviour of DDLPS.

The clinical presentation of DDLPS is usually vague and focused according to its anatomical location, accompanied by symptoms that do not manifest until the tumour reaches a considerable size and manages to affect adjacent structures. In the case of DDLPS located in the retroperitoneum, patients may present with intestinal alterations, urinary alterations, peripheral edema, the appearance of a palpable mass in the abdomen, involuntary weight loss and in some cases pain [10].

To make the diagnosis of retroperitoneal sarcomas, ultrasound can be used for an initial evaluation and detection of the presence of an abnormal mass in the peritoneal region [11]. Matthyssens *et al* [11], but the test of choice is contrast-enhanced computed tomography or magnetic resonance imaging of the abdomen and/or pelvis, in which LPSs are seen to be composed of a large adipose element [11, 12].

In these tumours, total surgical resection remains the treatment of choice [12]. However, a recent trial demonstrated that the combined treatment of preoperative radiotherapy with total surgical resection improved the prognosis of local recurrence in patients with retroperitoneal LPS compared with the use of only complete surgical resection [13]. However, this procedure is not usually definitive, since more than 80% of patients present with local recurrence of the tumour. The theory that supports these data is that the location of the tumour in the retroperitoneal region and the type of cells it attacks, particularly if it is visceral adipose tissue, makes it difficult to establish with precision the margins and the extension of the tumour, which makes it more probable that the surgical resection of the tumour is not total, facilitating the expansion of the tumour and making its complete control difficult [14].

The reported patient had no obvious symptomatology during the finding of the tumour recurrence; the tumour was discovered accidentally during a routine abdominal ultrasound. The location of our patient's current tumour is retroperitoneal and is classified as a high-grade DDLPS. In this case, although its histologic subtype predisposes it to recurrence, the patient's time to recurrence is more than 10 years, an interval longer than the expected average. In this case, the primary tumour behaved as a tumour of low histologic grade, although this is not usual for these types of tumours [15]. Subsequently, the recurrence was characterised as a tumour of high histological grade, which generates great questions regarding the biological behaviour of the tumour and prognosis of the patient in these cases, which is why further research in the anatomopathological and molecular field of these tumours is necessary.

## Conclusion

DDLPS is characterised by relapses in more than 80% of the patients who have it in a time not exceeding 5 years; however, we note that there is the possibility of presenting cases where the disease returns in a much longer time than expected, giving way to new questions regarding prognosis, survival and biological structure, are there new mutations or factors that contribute to this new time of recurrence, it is certainly something that needs to be studied. Detection and continuous surveillance of those patients who are at high risk of presenting the disease is essential to achieve an early diagnosis in time that may allow timely treatment.

## Conflicts of interest

There are no conflicts of interest.

## Funding

None of the above authors received financial support for this publication.

## Author contributions

All authors wrote, reviewed and read the entire article.
